# Beyond capability frameworks: Australia’s next challenge in preparing the digital health workforce

**DOI:** 10.3389/frhs.2026.1968499

**Published:** 2026-10-21

**Authors:** Salma Arabi, Kerryn Butler-Henderson

**Affiliations:** School of Nursing, Paramedicine and Healthcare Sciences, Charles Sturt University, Wagga Wagga, NSW, Australia

**Keywords:** digital capability, digital health, digital literacy, workforce development, workforce preparedness

## Abstract

Australia has made substantial progress in digital health workforce development through the establishment of nationally coordinated strategies, profession-specific capability frameworks, and the Australian Digital Health Capability Framework. Collectively, these initiatives have repositioned digital health from a specialised technical domain to a core professional competency expected across the health workforce. However, while considerable effort has focused on defining the capabilities required for contemporary digital practice, less attention has been directed towards ensuring workforce readiness. This perspective argues that capability and readiness are related but distinct concepts. Capability frameworks describe the knowledge, skills, and behaviours expected of health professionals, whereas readiness reflects the ability to apply these capabilities confidently, ethically, and adaptively within rapidly evolving healthcare environments. As digital health systems become increasingly influenced by artificial intelligence, predictive analytics, and data-driven decision-making, workforce preparedness will depend on more than technical competence alone. It will require critical thinking, ethical reasoning, digital and data literacies, and the capacity to work effectively alongside emerging technologies. We propose that, while capability frameworks will continue to evolve alongside technological and regulatory change, Australia's next challenge is ensuring that existing capability expectations are translated into educational practice, workplace learning, professional development, and measurable workforce outcomes. Sustained implementation, evaluation, and collaboration will be essential to ensure a future-ready digital health workforce.

## 1 Introduction

Digital health and virtual care have become integral components of contemporary healthcare delivery, transforming how health professionals communicate, make clinical decisions, access information, and partner with consumers and carers. The rapid expansions of telehealth, electronic health records (EHRs), remote monitoring, clinical decision-support systems, and artificial intelligence (AI) have accelerated the digital transformation of healthcare globally. However, evidence suggests that the benefits of digital health technologies are not realised through technological implementation alone; rather, successful adoption depends heavily on the preparedness and capability of the health workforce to use digital tools effectively, safely, and ethically (1). Studies consistently identify workforce capability as a critical enabler of digital transformation and a determinant of technology adoption, quality of care, and patient outcomes (2, 3).

Despite growing recognition of the importance of digital health capability, significant variability remains in digital competence across the health professions. While digital health capability frameworks and educational resources are becoming increasingly available, implementation across education and practice settings remains uneven (4). Interventions designed to improve digital readiness, and capability can strengthen workforce confidence and skills, but evidence for sustained organisational and workforce-level outcomes remains limited (4). Many competency requirements have yet to be systematically embedded within health professional curricula (5). This highlights a persistent gap between defining the capabilities required for digital practice and ensuring that the workforce is consistently prepared to meet them.

Australia has been proactive in responding to these challenges through the development of coordinated digital health workforce agenda. The National Digital Health Workforce and Education Roadmap (6) highlighted the need for a nationally consistent approach to capability development and positioned digital capability as essential for contemporary healthcare practice. This work subsequently informed the development of several profession-specific and national frameworks, including the National Nursing and Midwifery Digital Health Capability Framework (7), the Digital Health in Medicine Capability Framework (8), and the Digital Health Capability Framework for Allied Health Professionals (9), and more recently the Australian Digital Health Capability Framework (10). Collectively, these initiatives have shifted digital health from being viewed as a specialised technical skill toward recognition as a core professional competency expected across the health workforce.

This perspective brings together insights from major Australian digital health workforce initiatives, national capability frameworks, and selected literature to provide a contemporary view of workforce development priorities and opportunities.

## 2 Australia’s digital health capability success story

Over the past decade, Australia has undertaken a significant transformation in its approach to digital health workforce development. Rather than positioning digital health as a specialised area of practice or an optional technical skill, national policy and professional initiatives have increasingly recognised digital capability as a core component of contemporary healthcare practice (6, 11). This shift reflects growing acknowledgement that safe, effective, and person-centred care is now inextricably linked to the ability of health professionals to work confidently within digitally enabled healthcare environments (12, 13). Australia’s commitment to developing a digitally capable workforce has been further reinforced through national strategies and workforce development initiatives that recognise workforce capability as a critical enabler of digital health transformation.

A major milestone in this evolution was the development of the Australian Digital Health Capability Framework (10), which provides a shared, cross-disciplinary articulation of the knowledge, skills, and behaviours required across the health workforce. Developed to support the goals of Australia’s National Digital Health Strategy (11), the framework establishes digital capability as relevant to all members of the health and care workforce, regardless of profession or career stage. Importantly, it moves beyond a narrow focus on technology-use to encompass broader domains including digital professionalism, leadership and advocacy, and data and information quality. In doing so, the framework positions digital capability as a foundational requirement for contemporary practice rather than discipline-specific or technical competencies.

The Australian Digital Health Capability Framework (10) sits alongside a suite of discipline-specific capability frameworks that have collectively contributed to the maturation of Australia’s digital health workforce agenda. In nursing and midwifery, digital capability is articulated through the National Nursing and Midwifery Digital Health Capability Framework (7), while the Capability Framework on Digital Health in Medicine (8) and the Digital Health Capability Framework for Allied Health Professionals (9) provide similar guidance for their respective disciplines. Although developed for different professional contexts, these frameworks demonstrate considerable alignment in their emphasis on digital professionalism, information and data literacy, technology-enabled care, human-centred practice, and the safe use of digital technologies. This convergence is noteworthy, reflecting an emerging national consensus regarding the capabilities required for contemporary healthcare practice and signalling that digital capability is increasingly viewed as a shared professional expectation across the workforce.

Importantly, efforts have increasingly extended beyond framework development to support implementation. The recently launched Digital Health Train-the-Trainer Toolkit (14), developed by the Australian Digital Health Agency in partnership with the Australian Council of Senior Academic Leaders in Digital Health, provides practical resources to assist university educators in embedded eight digital health learning outcomes within pre-registration health degrees. Its development recognises that defining capability expectations alone is insufficient and that educator capability is a critical determinant of successful curriculum integration.

Taken together, these examples represent a significant achievement in health workforce development. While several countries have developed national digital health workforce initiatives and capability frameworks, Australia has established a particularly coordinated ecosystem spanning workforce strategy, discipline-specific capability expectations, pre-registration preparedness, and implementation supports. Australia’s most important accomplishment has been the successful repositioning of digital health capability from a supplementary technical skill to a recognised professional competency that is increasingly embedded within expectations of education, workforce development, accreditation, and clinical practice. However, while Australia has made considerable progress in defining what digitally capable practice should look like, the more difficult challenge lies in ensuring that these expectations are consistently translated into educational outcomes, workforce behaviours and readiness.

## 3 Capability frameworks are necessary but not sufficient

Australia’s digital health capability agenda has reached a level of maturity where the discussion can move beyond questions of definition. There is now considerable agreement regarding the knowledge, skills, and behaviours required for safe and responsible digital practice. The more challenging question is whether clearly articulated capability expectations are translating into workforce readiness.

Viewed through this lens, recent initiatives such as the Digital Health Train-the-Trainer Toolkit (14) are particularly significant. Rather than further refining capability statements, they focus on supporting educators to embed digital health within pre-registration curricula. Such initiatives implicitly acknowledge that capability frameworks alone do not produce a digitally capable workforce. Their value is realised only when translated into training, assessment, simulation, work-integrated learning, and professional development activities that enable learners to engage with digital technologies in authentic practice contexts.

The distinction between capability, competence, performance and readiness warrants greater attention. Capability frameworks describe the knowledge, skills, and behaviours expected of health professionals (10). Competence refers to the possession of these capabilities at an appropriate standard, while performance reflects their demonstrated application in practice (15). Workforce readiness extends beyond both concepts and may be understood as the ability to apply digital health knowledge, skills, and behaviours safely, effectively, ethically, confidently, and adaptively within authentic practice environments. Readiness reflects the extent to which individuals are prepared to translate capability into performance within complex and evolving healthcare settings. It therefore encompasses not only competence, but also judgement, confidence, contextual awareness, and adaptability (16, 17). Workforce readiness may also be viewed through the lens of Miller’s pyramid, with readiness reflected in the ability to translate knowledge and competence into effective performance within authentic practice settings (15). Reviews of digital readiness interventions continue to report variation in educational approaches, implementation strategies, and measures of success, suggesting that capability development is influenced as much by educational and organisational contexts as by the frameworks themselves (18).

From this perspective, Australia’s immediate workforce challenge is less about developing new frameworks and more about ensuring that existing frameworks are effectively translated into education, practice, and workforce development. While frameworks will continue to evolve alongside technological and regulatory change, greater attention is now needed for implementation and evaluation. The success of the country’s digital health capability agenda will ultimately be determined not by the number of frameworks produced but by the extent to which their expectations become observable in educational outcomes, professional practice, and workforce readiness.

## 4 From capability to workforce readiness

If capability frameworks describe what health professionals should know and be able to do, workforce readiness requires consideration of the broader conditions that enable those capabilities to be enacted in practice. Readiness is not simply the accumulation of technical skills. Rather it reflects the extent to which individuals can apply knowledge, exercise professional judgement, adapt to changing circumstances, and engage safely and responsibly with technologies. From this perspective, digital capability should be viewed as a necessary foundation for workforce preparedness, but not its endpoint.

Workforce readiness is also shaped by organisational readiness. Leadership support, digital infrastructure, interoperability, governance arrangements, workflow integration, technical support, workplace culture, and protected learning opportunities all influence whether digital capabilities can be translated into practice. Consequently, readiness should be viewed not only as an individual workforce attribute but also as a product of supportive organisational environments that enable safe and effective digital practice.

Consideration must also be given to equity in workforce readiness initiatives. Digital capability development and implementation experiences are likely to vary across metropolitan, regional, rural, and remote settings, professional groups, and career stages. Differences in digital infrastructure, access to development opportunities and prior experience with digital technologies may influence readiness and should be considered when designing workforce development strategies.

This distinction is particularly important given the increasingly complex digital health environment. Health professionals are now expected not only to use digital technologies but also to critically appraise information, interpret data, protect privacy, navigate ethical dilemmas, support consumer engagement, and respond to emerging technologies such as AI. In AI-enabled environments, readiness additionally requires the ability to evaluate the reliability of AI-generated outputs, recognise automation bias and hallucinated content, understand the limitations of algorithmic recommendations, and exercise professional judgement when using AI-supported tools. These expectations extend beyond traditional conceptions of technical competence and require a broader set of professional capabilities that can be adapted to diverse and evolving contexts.

Achieving workforce readiness also requires infrastructure that supports implementation, evaluation, and continuous improvement. The Australian Council of Senior Academic Leaders in Digital Health reflects this broader systems approach through its focus on both education and workforce development, and the advancement of digital health research (19). This recognises that workforce preparedness depends not only on embedding digital capabilities within curricula or on the job training, but also on building the evidence base needed to evaluate, refine, and sustain workforce development initiatives over time. Complementing these efforts, the DHWorx (20), an Australian and New Zealand digital health workforce research consortium, provides a mechanism for collaboration and scholarship focused on understanding workforce capability, preparedness, and emerging educational needs. The distinction between capability and readiness can be illustrated through practical examples. As shown in Table 1, capability domains can be translated into observable indicators of workforce readiness that reflect the application of knowledge, skills, and professional judgement in practice. These examples are illustrative only and demonstrate how capability expectations may be translated into observable indicators of workforce readiness.

**Table 1:** Illustrative examples of translating digital health capability domains into workforce readiness indicators.

Australian Digital Health Capability Framework domain (10)	Capability statement	Example workforce readiness indicator
Domain A: Digital professionalism	A2.3 Maintains awareness of cultural, ethical, and socioeconomic issues when accessing and using digital health information and uses the information respectfully	Applies professional judgement when digital technologies create inequities, uncertainty, or ethical dilemmas, including access, privacy, security, communication and AI-related issues.
Domain B: Leadership and advocacy	B3.1 Promotes cross-sector collaboration in design, implementation and evaluation of emerging and innovative digital health technologies	Participates effectively in multidisciplinary and cross-sector collaboration to support the design, implementation, and evaluation of digital health technologies.
Domain C: Data and information quality	C2.1 Uses relevant data and information to support own decision-making, within scope of role and healthcare setting	Critically evaluates data and AI-generated outputs and incorporates them appropriately into clinical decision-making
Domain D: Information enabled care and services	D2.3 Able to support patients/consumers to confidently access and evaluate electronic health information and digital services	Supports patients and consumers with diverse needs and levels of digital literacy to confidently access, navigate, and evaluate electronic health information and digital health services.
Domain E: Technology	E3.3 Understands where to seek support and how to work around technology or process failures to maintain safe service delivery	Recognises technology or process failures, seeks appropriate support, and uses safe alternative processes to maintain continuity and safety of service delivery.

Looking forward, emphasis should shift from developing additional frameworks towards strengthening the educational, organisational, and regulatory mechanisms that enable workforce readiness. This includes supporting educators to integrate digital health throughout curricula, developing robust approaches to assessment, creating opportunities for authentic workplace learning, and ensuring that capability development continues across the professional lifespan. Equally important will be fostering critical thinking, ethical reasoning, and adaptability to ensure that health professionals remain capable of navigating technologies that continue to evolve long after graduation.

Translating capability frameworks into workforce readiness requires alignment across education, professional development, and workplace learning environments. Capability expectations can inform curriculum design, experiential learning opportunities, continuing professional development activities, and workplace support strategies, creating multiple opportunities for health professionals to develop and apply digital health capabilities throughout their careers.

## 5 Discussion

Australia’s digital health capability journey demonstrates that national coordination can shift digital health from a specialist or optional skill set to a shared expectation of contemporary health professional practice. The sequence from the National Digital Health Workforce and Education Roadmap to discipline-specific frameworks and the Australian Digital Health Capability Framework has created a coherent language for describing expected capabilities across professions (1, 6–10). This is an important achievement because a digitally enabled workforce is now recognised as essential to safe, effective, person-centred care, yet Australian commentators continue to identify gaps between policy ambition, health professional education and implementation in practice (13). The next stage is therefore less about defining capability and more about ensuring that capability is developed, assessed, and enacted across education, clinical settings and continuing professional development.

As argued throughout this paper, workforce readiness extends beyond capability acquisition to encompass the application of digital capabilities within authentic clinical contexts. Capability frameworks describe what professionals should know and be able to do, whereas readiness requires confidence, judgement, and the ability to apply capability in authentic and changing clinical contexts (14, 15, 21). This distinction is reinforced by evidence that educational interventions can improve digital competence, but approaches, outcomes and measures remain variable (16). Similarly, international consensus work in medical education has emphasised that digital health curricula must be systematically designed around professionalism, patient and population digital health, health information systems and health data science, rather than treated as isolated technical content (22). For Australia, this suggests that framework alignment is only the first step; the more difficult task is curriculum integration, valid assessment and workplace translation.

Workforce preparedness is multidimensional. Digital health practice involves interpretation, evaluation, ethical reasoning, communication, and partnership with stakeholders, not simply the operation of systems. Recent work on digital health readiness similarly argues for assessment approaches that consider people’s goals, motivations, literacy, aptitude and context, rather than assuming access or exposure equates to readiness (17). Applied to workforce development, this reinforces the need to prepare health professionals to act as critical, reflective, and adaptive users of technology. This becomes more urgent as AI, predictive analytics, and increasingly autonomous digital tools become embedded in healthcare. Emerging literature cautions that AI should be positioned as a means of augmenting the health workforce rather than replacing professional judgement, with responsible innovation requiring ethical governance, human-centred design and workforce leadership (23). Readiness is connected to organisational openness to change and that training should be planned as part of institutional implementation, not left to individual enthusiasm (24). Australia’s capability agenda therefore needs to evolve from digital tool proficiency towards readiness for digitally mediated decision-making, including critical appraisal of AI outputs, data stewardship, privacy, accountability, bias awareness, and explainability. Importantly, workforce readiness is influenced by organisational conditions as much as individual capability development, reinforcing the need for implementation strategies that span education, workplaces, and health systems.

Initiatives such as the Digital Health Train-the-Trainer Toolkit, the Australian Council of Senior Academic Leaders in Digital Health, DHWorx, and the Australian Alliance for AI in Healthcare show that Australia is beginning to build the implementation infrastructure required to move from framework development to workforce preparedness (19, 20). The priority now is sustained evaluation: whether digital health capabilities are embedded across pre-registration curricula, whether educators are capable and supported to teach and assess these capabilities, whether simulation and workplace learning offers authentic experiences, and whether graduates and practising clinicians can demonstrate safe, responsible, and adaptive digital practice over time. Without this evidence, capability frameworks risk remaining aspirational documents rather than mechanisms for workforce transformation.

Australia’s digital health capability journey provides a strong foundation for workforce preparedness. Future efforts should focus on implementation, evaluation, and sustained workforce development to ensure capability expectations are consistently translated into practice. The mature ecosystem of strategies, frameworks, and educational resources that define what digitally capable practice should look like now needs to focus on readiness: building the confidence, judgement, literacies, and adaptability required to practise safely in digitally enabled and AI-augmented health systems. This will require coordinated action across universities, accreditation bodies, health services, professional organisations, policymakers and researchers. If Australia can connect capability expectations with evidence-informed implementation and continuous evaluation, it will be well placed to move beyond defining the digitally capable workforce towards producing and sustaining it.

This perspective focuses primarily on the Australian context and draws on selected literature and national initiatives rather than a systematic review of international evidence. Empirical evidence regarding the long-term workforce impact of digital health capability frameworks remains limited, and readiness requirements are likely to vary across professions, career stages, organisations, and healthcare settings. In addition, the rapid evolution of digital technologies, AI-enabled tools, and regulatory environments means that capability frameworks and associated readiness expectations will require ongoing refinement over time.

## Data Availability

The original contributions presented in the study are included in the article/Supplementary Material, further inquiries can be directed to the corresponding author.
